# Marital status and all-cause mortality rate in older adults: a population-based prospective cohort study

**DOI:** 10.1186/s12877-023-03880-8

**Published:** 2023-04-04

**Authors:** Lei Wang, Zhong Yi

**Affiliations:** 1 Department of Cardiology, Aerospace Center Hospital, 15 Yuquan Road, Haidian District, 100049 Beijing, PR China; 2 Department of Geriatric Medicine, Aerospace Center Hospital, 15 Yuquan Road, Haidian District, 100049 Beijing, PR China

**Keywords:** Marital status, All-cause mortality, Older adults

## Abstract

**Background:**

Living with a partner and separation is becoming more common among older people. Mortality disparities associated with marital status are significant in increasingly diverse aging populations. The link between marital status and all-cause mortality risk in older adults remains uncertain.

**Methods:**

This prospective cohort study included data from the US National Health and Nutrition Examination Survey (NHANES). We included NHANES participants ≥ 60 years of age (data from 1999 to 2014). Data for mortality follow-up beginning from the commencement date of survey participation to the last day of December 2015. Univariate- and multivariate-adjusted Cox proportional hazard models for marital status were estimated, and the findings were presented as regression coefficients and 95% confidence intervals (CI). Kaplan–Meier curves were reported.

**Results:**

Compared to never married individuals, the risk of all-cause mortality was 0.77 (0.50–1.18), 0.72 (0.56–0.93), 0.56 (0.36–0.88), and 0.84 (0.67–1.07) in those people living with a partner, married, separated, and divorced, respectively, after adjusting for demographics, socioeconomics, behavior, anthropometric variables, and medical history. The risk of all-cause mortality was 1.24 (0.97–1.59) in widowed participants.

**Conclusion:**

This population-based cohort study included a large sample size followed by long-term follow-up. The association between marriage, health, and reduced mortality in older individuals has been illustrated in this study. Being married or separated was associated with a lower risk of mortality.

**Supplementary Information:**

The online version contains supplementary material available at 10.1186/s12877-023-03880-8.

## Background

Marriage has been shown to have a significant and beneficial impact on human survival over the course of many decades [1, 2]. According to the majority of research, those who are married have a decreased death rate as opposed to single people [3, 4]. The protective impact of marriage ought not to be ignored; nonetheless, this association may be partly due to a selection effect for individuals who opt into marriage. In contrast with people who are not married, married people tend to have greater economic resources, greater social support networks, a higher standard of living, healthier lives and behaviors, and access to better medical treatment, which has led many people to believe that marital status is a viable indicator of social support [5, 6].

Mortality disparities associated with marital status are significant in increasingly diverse aging populations [3]. Living with a partner and separation is becoming more common among older people [7, 8]. However, these investigations cannot uncover any differences between unmarried people and separated people [9]. Even though numerous earlier research has shown differences between married and single individuals [10, 11], but sex differences are yet unclear [12, 13]. Previous studies speculated that married men benefited from their spouses, who helped them maintain a healthier lifestyle and curtail detrimental habits [14–16]. Therefore, these past researches need to be updated. The differences in mortality rates between marriage types and sex need further study.

This research determined whether there is an association between older adults’ marital status and their risk of any-cause death. To conduct a more in-depth study and investigate the factors that impact one’s probability of survival, we focused on various marital statuses, such as being single, living with a partner, married, divorced, widowed, or separated.

## Methods

### Ethics statement

We confirmed that all methods were carried out in accordance with relevant guidelines and regulations. The data for this study were acquired from the National Health and Nutrition Examination Survey in the United States (NHANES). The de-identified data are freely available on the NHANES website (https://www.cdc.gov/nchs/nhanes.htm). We confirmed that informed consent was obtained from all subjects and/or their legal guardian(s). We confirmed that all experimental protocols were approved by The National Center for Health Statistics (NCHS) Research Ethics Review Board.

### Study design and population

The present prospective cohort study included data from the US National Health and Nutrition Examination Survey (NHANES) from 1999 to 2014. We recruited NHANES participants ≥ 60 years of age. The US NHANES adopted a multistage and comprehensive probability sampling method to collect health data representing the US population. Data were collected via in-person interviews, mobile physical examinations, and laboratory tests. Details of the NHANES Laboratory/Medical Technologists and Anthropometry Procedures are illustrated in a previous study [17].

### Baseline data collection

Information on covariates was obtained using baseline questionnaires. These questionnaires contained the following information: age, sex, marital status, family income-poverty ratio, education level, race/ethnicity, smoking status, drinking status, and a self-reported baseline medical history. The self-reported medical history included common diseases (diabetes, myocardial infarction, hypertension, hypercholesterolemia, cardiovascular disease, stroke, and chronic bronchitis) and medications (antihypertensives, hypoglycemic agents, and lipid-lowering medications). Body mass index (BMI) was computed premised on the participant’s weight and height weight metrics. Laboratory measurements were done per the laboratory procedure manual for NHANES. The NHANES guidelines (https://wwwn.cdc.gov/nchs/nhanes/analyticguidelines.aspx) describe the methodologies and processes utilized for clinical laboratory data and study visits.

### Marital status

The NHANES collected data on the marital status of each member of the cohort. Six marital status categories were used: never married, cohabiting with a partner, married, separated, divorced, or widowed.

Diagnostic criteria for smoking were as follows: never smoked (smoked < 100 cigarettes in his life), former smoker (smoked > 100 cigarettes in their life and are no longer smoking), smoker (smoked > 100 cigarettes in their life and smokes sometimes or every day).

Diagnostic criteria for alcohol use were as follows: current heavy alcohol user (≥ 4 drinks per day for males, ≥ 3 drinks per day for females, or ≥ 5 days per month of binge drinking), current moderate alcohol user (≥ 3 drinks per day for males, ≥ 2 drinks per day for females, or ≥ 2 days per month of binge drinking) or current mild alcohol user (not meet the above) [18].

The diagnostic criteria for diabetes were [19]: the patient self-reported diabetes, use of diabetes medication or insulin, hemoglobin A1c (HbA1c) level ≥ 6.5%, random plasma blood glucose ≥ 11.1 mmol/l (200 mg/dl), fasting plasma glucose ≥ 7.0 mmol/l (126 mg/dl), and/or two-hour oral glucose tolerance test (OGTT) blood glucose ≥ 11.1 mmol/l(200 mg/dl).

The diagnostic criteria for hypertension were [20, 21]: the patient self-reported hypertension, use of antihypertensive medication, and/or average blood pressure (diastolic blood pressure ≥ 90mmHg and/or systolic blood pressure ≥ 140mmHg). To calculate the average blood pressure, the diastolic reading with zero was not applicable to the calculation of the diastolic average. If all diastolic readings were zero, then the average would be zero. If only one blood pressure reading was obtained, that reading was registered as the average. If there was more than one blood pressure reading, the first reading was excluded from the average.

The prevalence of cardiovascular disease (CVD) among participants was evaluated based on whether or not they had self-reported a diagnostic assessment of a minimum of one of the following five CVD subsets: congestive heart failure (CHF), angina, myocardial infarction, coronary artery disease (CAD), and/or stroke. Self-reported affirmative response (yes or no) for a minimum of one of these criteria was used to determine the occurrence of CVD, and participants who had CVD could belong to more than one subtype of CVD.

Hyperlipidemia was defined as dyslipidemia according to the 2019 ESC/EAS guidelines [22].

Chronic kidney disease (CKD) was described as aberrations in renal function according to the KDIGO 2021 Clinical Practice Guideline [23].

### Mortality

The NHANES-assigned sequence number was used to link de-identified and anonymized participant data to longitudinal Medicare and mortality data. The mortality follow-up data include the period beginning from the first day of survey participation to the last day of December 2015. All-cause mortality included death from heart diseases (I00–I09, I11, I13, I20–I51), diabetes mellitus (E10–E14), malignant neoplasms (C00–C97), Alzheimer’s disease (G30), cerebrovascular diseases (I60–I69), chronic lower respiratory diseases (J40–J47), and other causes [24]. The 10th version of the International Classification of Diseases was used to establish the cause of death.

### Statistical analysis

Continuous data were reported as mean ± standard deviation (SD; Gaussian distribution) or median (range; Skewed distribution), and categorical data were reported as numbers (%). χ2 (categorical variable), one-way ANOVA test (normal distribution), or Kruskal–Wallis H test (skewed distribution) were used to examine differences in marital status.

Before data analysis, variables were inspected for missing values. The proportion of missing data was 0.00-85.3% (alcohol use). To include these data from the analyses, dummy variables were used to indicate missing covariate values [25].

In addition, univariate- and multivariate-adjusted Cox proportional hazard models were estimated for marital status, and the findings were presented as regression coefficients and 95% confidence intervals (CI). Model 1 unadjusted. Model 2 adjusted for sex. Model 3 additionally adjusted for age, ethnicity, education level, family income-poverty ratio and BMI level. In model 4, we additionally adjusted for smoking status, alcohol drinks, diabetes, hypertension, hyperlipidemia, cardiovascular disease and chronic kidney disease. Estimation of survival over time progression was performed with Kaplan–Meier curves, and the log-rank test was applied to evaluate the differences in outcomes demonstrated by the various survival curves.

### Sensitivity analysis

Univariate- and multivariate-adjusted Cox proportional hazard models were estimated for marital status in the different sex and different age categories. The findings were presented as regression coefficients and 95% confidence intervals (CI).

The study utilized the statistical package R (The R Foundation; http://www.r-project.org; version 4.1.2) to perform all statistical analyses that were modified for complex survey design and population weighting per survey protocols. The findings may be applied and extrapolated to the entire United States adult population by incorporating population weights, stratum variables, and main sampling units into the analysis, accounting for differential probability of inclusion into the sample and non-response bias.

## Results

The 82,091 NHANES participants represented 442.2 million non-institutionalized residents of the United States. A total of 15,036 older adults (represented 75.2 million; aged 70.6 ± 7.4 years; 56.0% women, 80.0% non-Hispanic White people, 8.6% non-Hispanic Black people, and 3.6% Mexican Americans) were included in the study. Lack of marital status differs between males and females (Table S1).

Table 1 provides weighted baseline features of research subjects stratified by marital status. A significant age difference was observed between marital status (P < 0.001), as widowed people were older (75.6 ± 7.0 years). Additionally, a higher proportion of widows were women (81.7%, P < 0.001). The majority of the non-Hispanic White people were married (83.4%, P < 0.001). The participants with higher family income-poverty ratio levels were the married participants (P < 0.001). Most participants with a “college or above” level of education were found to be divorced (49.6%, P < 0.001). The differences observed for CVD was probably related to sex (P < 0.001) (Table S2).

**Table 1:** Weighted features of the research subjects by marital status

	Level	Overall	Never married	Living with partner	Married	Divorced	Separated	Widowed	*p*
N		73,772,340	2,539,091	1,154,684	44,872,600	7,452,897	878,223	16,874,845	
Age		70.6 (7.4)	68.6 (7.0)	66.9 (6.2)	69.4 (6.8)	67.8 (6.2)	68.3 (6.5)	75.6 (7.0)	< 0.001
Sex (%)	Female	41,019,469 (55.6)	1,376,515 (54.2)	493,880 (42.8)	19,983,311 (44.5)	4,833,751 (64.9)	543,697 (61.9)	13,788,316 (81.7)	< 0.001
	Male	32,752,871 (44.4)	1,162,576 (45.8)	660,804 (57.2)	24,889,289 (55.5)	2,619,146 (35.1)	334,527 (38.1)	3,086,529 (18.3)	
Race/ethnicity (%)	Non-Hispanic White	58,978,841 (79.9)	1,684,197 (66.3)	861,299 (74.6)	37,444,070 (83.4)	5,548,333 (74.4)	260,183 (29.6)	13,180,759 (78.1)	< 0.001
	Non-Hispanic Black	6,341,206 (8.6)	455,687 (17.9)	124,787 (10.8)	2,463,584 (5.5)	1,061,988 (14.2)	345,487 (39.3)	1,889,673 (11.2)	
	Mexican American	2,645,516 (3.6)	96,991 (3.8)	62,663 (5.4)	1,542,661 (3.4)	219,267 (2.9)	106,228 (12.1)	617,706 (3.7)	
	Other Race	5,806,777 (7.9)	302,216 (11.9)	105,935 (9.2)	3,422,285 (7.6)	623,309 (8.4)	166,325 (18.9)	1,186,707 (7.0)	
Education (%)	College or above	34,458,533 (46.7)	1,077,586 (42.4)	566,037 (49.0)	23,179,427 (51.7)	3,692,918 (49.6)	259,184 (29.5)	5,683,380 (33.7)	< 0.001
	High school or equivalent	19,594,991 (26.6)	629,508 (24.8)	225,046 (19.5)	11,789,518 (26.3)	1,888,617 (25.3)	145,721 (16.6)	4,916,581 (29.1)	
	Less than high school	19,582,903 (26.5)	812,461 (32.0)	363,600 (31.5)	9,864,904 (22.0)	1,857,497 (24.9)	470,465 (53.6)	6,213,976 (36.8)	
Family income-poverty ratio		2.85 (1.55)	2.23 (1.55)	2.58 (1.56)	3.23 (1.49)	2.39 (1.52)	1.80 (1.42)	2.19 (1.40)	< 0.001
BMI (kg/m^2^)		28.57 (5.89)	29.30 (7.49)	28.49 (6.76)	28.65 (5.52)	28.62 (6.55)	29.33 (7.09)	28.24 (6.15)	0.15
Smoking status (%)	Never	35,343,436 (47.9)	1,311,996 (51.7)	317,148 (27.5)	21,291,721 (47.4)	2,707,509 (36.3)	473,250 (53.9)	9,241,812 (54.8)	< 0.001
	Former	29,861,685 (40.5)	796,069 (31.4)	461,701 (40.0)	19,512,744 (43.5)	2,968,625 (39.8)	232,992 (26.5)	5,889,554 (34.9)	
	Now	8,529,651 (11.6)	428,889 (16.9)	375,835 (32.5)	4,058,098 (9.0)	1,776,764 (23.8)	169,128 (19.3)	1,720,939 (10.2)	
Alcohol drinks (%)	Mild	1,550,418 (2.1)	41,938 (1.7)	47,388 (4.1)	1,218,941 (2.7)	99,162 (1.3)	4358 (0.5)	138,631 (0.8)	< 0.001
	Moderate	7,023,283 (9.5)	247,764 (9.8)	224,868 (19.5)	4,280,787 (9.5)	1,061,036 (14.2)	32,517 (3.7)	1,176,311 (7.0)	
	Heavy	3,378,599 (4.6)	182,712 (7.2)	91,416 (7.9)	1,916,460 (4.3)	671,543 (9.0)	82,195 (9.4)	434,273 (2.6)	
Diabetes (%)	No	56,903,266 (75.6)	5,615,270 (75.3)	905,376 (78.4)	34,365,303 (76.6)	1,828,254 (72.0)	615,147 (70.0)	12,381,376 (73.4)	0.058
	Yes	18,061,614 (24.5)	1,837,627 (24.7)	249,308 (21.6)	10,507,297 (23.4)	710,837 (28.0)	263,076 (30.0)	4,493,469 (26.6)	
Hypertension (%)	No	31,074,432 (42.1)	1,149,781 (45.3)	523,141 (45.3)	19,875,100 (44.3)	3,252,832 (43.6)	327,261 (37.3)	5,946,315 (35.2)	< 0.001
	Yes	42,697,908 (57.9)	1,389,310 (54.7)	631,543 (54.7)	24,997,500 (55.7)	4,200,064 (56.4)	550,962 (62.7)	10,928,530 (64.8)	
Hyperlipidemia (%)	No	13,152,866 (17.8)	648,670 (25.5)	200,938 (17.4)	7,452,895 (16.6)	1,356,923 (18.2)	225,211 (25.6)	3,268,230 (19.4)	< 0.001
	Yes	60,617,684 (82.2)	1,890,421 (74.5)	953,746 (82.6)	37,419,705 (83.4)	6,095,974 (81.8)	653,013 (74.4)	13,604,826 (80.6)	
CVD (%)	No	55,494,033 (75.2)	2,015,061 (79.4)	857,433 (74.3)	34,430,791 (76.7)	5,856,535 (78.6)	661,761 (75.4)	11,672,452 (69.2)	< 0.001
	Yes	18,272,222 (24.8)	524,030 (20.6)	296,276 (25.7)	10,439,781 (23.3)	1,593,280 (21.4)	216,462 (24.6)	5,202,393 (30.8)	
CKD (%)	No	23,954,696 (32.5)	931,412 (36.7)	517,880 (44.9)	15,732,014 (35.1)	2,784,686 (37.4)	284,088 (32.3)	3,704,616 (22.0)	< 0.001
	Yes	12,797,560 (17.3)	426,802 (16.8)	191,790 (16.6)	6,795,109 (15.1)	1,426,716 (19.1)	153,491 (17.5)	3,803,652 (22.5)	

Data were presented as frequencies (percentages), mean (SD), or median (IQR).

Abbreviations:

CKD, chronic kidney disease

CVD, cardiovascular disease

BMI, body mass index. BMI is calculated as follows: the weight in kilograms (kg) / height in square meters (m^2^)

In the 84-month follow-up, 5031 deaths were reported, including 1002 deaths from cancer, 959 from heart disease, and 229 from cerebrovascular disease.

**Fig. 1:**
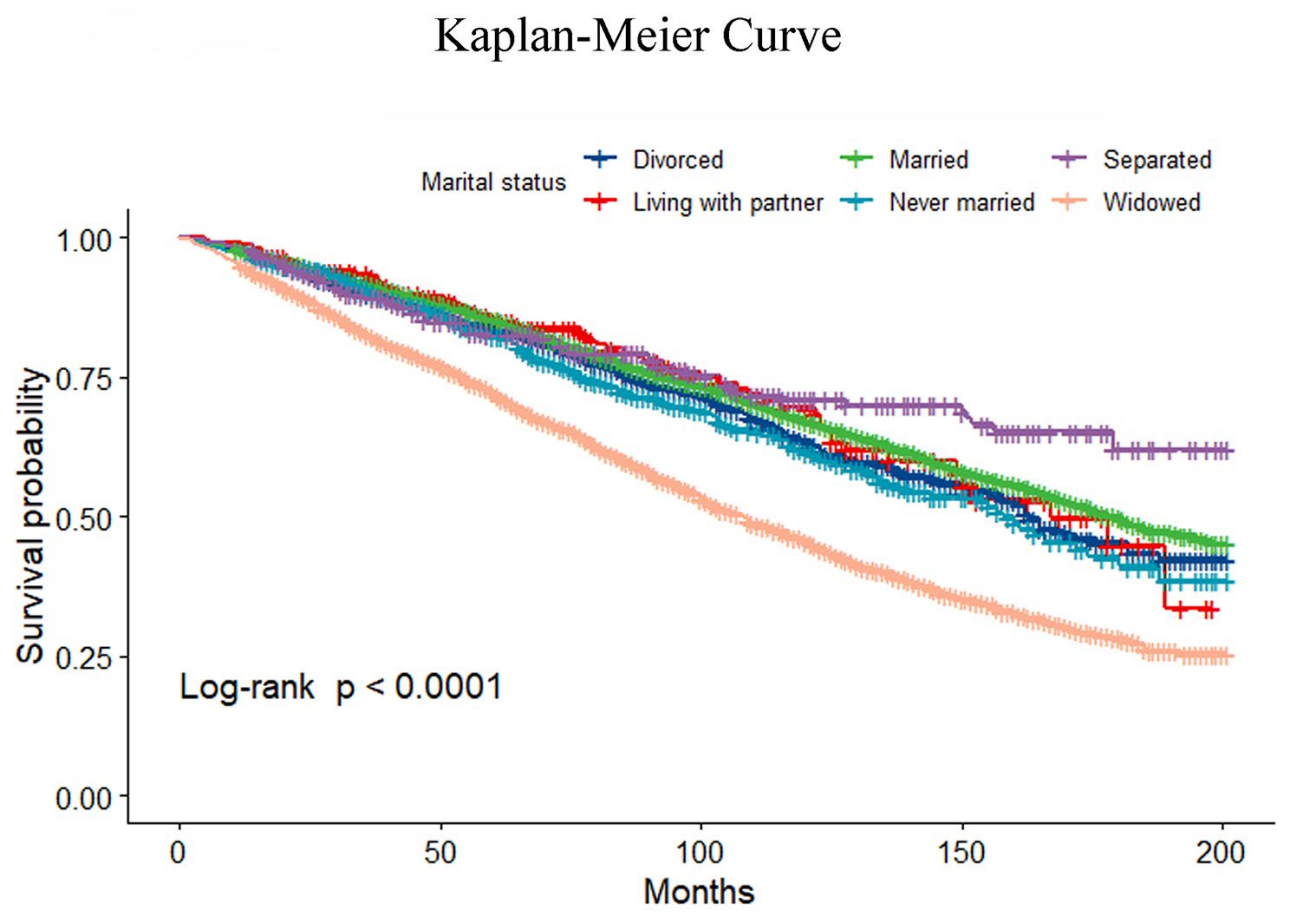
Kaplan–Meier curve of marital status

Table 2 provides the unadjusted and adjusted models for all-cause mortality. Within the unadjusted weighted model, compared to never married individuals, the risk of all-cause mortality was 0.78 (0.51–1.20), 0.65 (0.51–0.83), 0.58 (0.38–0.88), and 0.82 (0.64–1.05) in the participants living with a partner, married, separated, and divorced, respectively. The risk of all-cause mortality was 1.32 (1.05–1.66) in widowed participants (Table S3, Fig. 1). In model 4, compared to never married individuals, the risk of all-cause mortality was 0.77 (0.50–1.18), 0.72 (0.56–0.93), 0.56 (0.36–0.88), and 0.84 (0.67–1.07) in those participants living with a partner, married, separated, and divorced, respectively, after adjusting for demographic, socioeconomics, behavior, anthropometric variables, and medical history. The risk of all-cause mortality was 1.24 (0.97–1.59) in widowed participants.

**Table 2:** Weighted associations between marital status and all-cause mortality (weighted N = 73,772,340)

	Never married	Living with partner	Married	Separated	Divorced	Widowed
Model1	1	0.78(0.51–1.20)	0.65(0.51–0.83)	0.58(0.38–0.88)	0.82(0.64–1.05)	1.32(1.05–1.66)
Model2	1	0.75(0.50–1.14)	0.60(0.46–0.77)	0.60(0.40–0.91)	0.84(0.65–1.10)	1.50(1.18–1.90)
Model3	1	0.84(0.56–1.26)	0.75(0.58–0.96)	0.60(0.38–0.95)	0.90(0.70–1.15)	1.42(1.12–1.81)
Model4	1	0.77(0.50–1.18)	0.72(0.56–0.93)	0.56(0.36–0.88)	0.84(0.67–1.07)	1.24(0.97–1.59)

Data are hazard ratio (95% CI)

Model 1 unadjusted

Model 2 adjusted for sex

Model 3 adjusted for sex, age, ethnicity, education level, family income-poverty ratio and BMI level

Model 4 adjusted for model 3 covariates as well as smoking status, alcohol drinks, diabetes, hypertension, hyperlipidemia, cardiovascular disease and chronic kidney disease

Abbreviations:

CI stands for confidence interval

BMI, the body-mass index is determined as follows: the weight in kilograms (Kgs) / (height in square meters (m^2^)

### Sensitivity analysis

In the adjusted models, the risk of all-cause mortality was 0.77(0.55–1.56) and 0.83(0.47–1.46) in male married participants and separated participants, respectively. The risk of all-cause mortality was lower in female married and separated participants, in the adjusted models (hazard ratio, 0.68; 95% CI, 0.50–0.94 and 0.35; 95% CI, 0.20–0.59) (Table S4-5, Figure S1). The risk of all-cause mortality was 0.54(0.36–0.80) and 0.48(0.27–0.85) in the age group 60–69 years married participants and separated participants, respectively(Table S6).

## Discussion

This population-based cohort study included a large sample size followed by long-term follow-up. This study demonstrated the link between marriage, health, and lower mortality. Being married or separated was associated with a lower risk of mortality. Stratified analysis by sex showed that the risk of all-cause mortality was lower in the female married and separated participants.

Studies have shown that married people tend to have better health outcomes [26]. Marital quality [27] and family support [28] can have a significant impact on a person’s well-being and health [29]. Meanwhile the experience of separation confers risk for poor health outcomes [30, 31]. Our observations about separation stand in contrast to previous studies. Previously, the experience of separation can also lead to poor health outcomes, especially for those who become overly immersed in their experience or have a history of mental health issues [32]. However, at present, living a healthy lifestyle has become a common ambition, and people are now attempting to pay more attention to their health [33], especially separated participants [34]. Specifically, one may speculate that in the absence of a close and personal marital relationship, separation may increase the chances for family support [35] and a better treatment outcome [36]. Sex-specific differences may be due to sample size differences [37] and sex differences in health domains [38]. Future studies with more participants will be required to confirm these observations. Previous studies have shown widowhood was linked to an elevated mortality rate in older age for both men and women [39, 40]. This is consistent with the results of our study.

Adjustments in a marriage could be beneficial to a person’s health because of the effect it has on social-cognitive and emotional functions (such as a feeling of security), psychopathology, health-associated behaviors, and physiological processes (e.g., immune, cardiac, and neuroendocrine functioning) [27, 41]. The increased risk of death from any cause that is linked to married status is likely due to the interaction of a number of underlying physiological variables as well as stressors [5, 40].

In spite of the large number of research reports that have been conducted on this topic, very few efforts have been made to quantify the extent to which married status is linked to overall mortality in people of older age. In addition, it is not quite clear if this relationship remains stable or changes depending on a number of characteristics, such as gender [2, 3, 42]. The increasing diversity of partnership experiences, especially during the second half of life, highlights the importance of examining how unions can shape health and well-being [43]. Our study focused on a range of marital conditions, including unmarried, living with a partner, married, divorced, widowed, or separated, for a more comprehensive analysis.

### Limitations of the study

The present study has several limitations. First, we could not obtain information on clinical diagnoses of depressive disorder. Second, during the follow up period participants could change their marital status, and information about the length of the marriage was missing, as well as whether it was the first or a consecutive marriage. Third, we did not have available data on behavioral changes (nutrition, physical activity, sleep), United States region, urbanization, and number of children. Future studies should focus on the mechanisms that underlie the link between marital status and mortality. Notwithstanding the few methodological and conceptual constraints, the results of this study could be essential in supporting health care practitioners in accurately classifying people who are considered to be “at-risk,“ and they could be incorporated into the programs that are currently used to estimate the death risk for older adults.

## Conclusion

This is population-based cohort research. The study included a large sample size for long-term general-population follow-up. Our study demonstrated the link between marriage and health and lowered mortality in older individuals. Being married or separated was associated with a lower risk of mortality.

## Electronic supplementary material

Below is the link to the electronic supplementary material.

Table S1. Weighted baseline characteristics of participants: marital status and marital status missing (weighted N = 75249951).
Table S2. Weighted baseline characteristics of participants in the different sex (weighted N = 73772340).
Table S3. Weighted univariate cox regression model (weighted N = 73772340).
Table S4 Weighted association between marital status and all-cause mortality for male in the multivariate and crude analyses (weighted N = 32752871).
Table S5 Weighted association between marital status and all-cause mortality for female in the multivariate and crude analyses (weighted N = 41019469).
Table S6 Weighted association between marital status and all-cause mortality for the three different age categories (weighted N = 73772340).
Figure S1. Kaplan?Meier curves of marital status for female and male.

## Data Availability

The datasets used and/or analyzed during the current study are available from the corresponding author on reasonable request.
