# Supplementary material for: Marital status and all-cause mortality rate in older adults: a population-based prospective cohort study
Source: BMC Geriatr. 2023 Apr 4;23:214. doi: 10.1186/s12877-023-03880-8 (PMC10074686; doi:10.1186/s12877-023-03880-8)

Supplemental Material

## Table S1. Weighted baseline characteristics of participants: marital status and marital status missing (weighted N=75249951).

|  | Level | Marital status | Marital status missing | p |
| --- | --- | --- | --- | --- |
| N |  | 73772340 | 1477611 |  |
| Age (years) |  | 70.5 (7.4) | 71.9 (7.7) | 0.005 |
| Sex (%) | Female | 41019469 (55.6) | 1085626 (73.5) | <0.001 |
|  | Male | 32752871 (44.4) | 391985 (26.5) |  |
| Race/ethnicity (%) | Non-Hispanic Black | 6341206 (8.6) | 139272 (9.4) | 0.185 |
|  | Non-Hispanic White | 58978841 (79.9) | 1250358 (84.6) |  |
|  | Mexican American | 2645516 (3.6) | 49284 (3.3) |  |
|  | Other Race | 5806777 (7.9) | 38697 (2.6) |  |
| Education (%) | College or above | 572241 (38.7) | 34458533 (46.7) | 0.229 |
|  | High school or equivalent | 490495 (33.2) | 19594991 (26.6) |  |
|  | Less than high school | 384105 (26.0) | 19582903 (26.5) |  |
| Family income-poverty ratio |  | 2.85 (1.55) | 2.64 (1.46) | 0.377 |
| BMI (kg/m^2^) |  | 28.59 (5.89) | 27.97 (5.92) | 0.082 |
| Smoking status (%) | Never | 652519 (44.2) | 35343436 (47.9) | 0.624 |
|  | Former | 554170 (37.5) | 29861685 (40.5) |  |
|  | Now | 222290 (15.0) | 8529651 (11.6) |  |
| Alcohol drinks (%) | Mild | 26155 (1.8) | 1550418 (2.1) | 0.97 |
|  | Moderate | 123662 (8.4) | 7023283 (9.5) |  |
|  | Heavy | 64744 (4.4) | 3378599 (4.6) |  |
| Diabetes (%) | No | 18061614 (24.5) | 285072 (19.3) | 0.193 |
|  | Yes | 55710726 (75.5) | 1192539 (80.7) |  |
| Hypertension (%) | No | 31074432 (42.1) | 741888 (50.2) | 0.051 |
|  | Yes | 42697908 (57.9) | 735723 (49.8) |  |
| Hyperlipidemia (%) | No | 238005 (16.1) | 13152866 (17.8) | 0.587 |
|  | Yes | 1229480 (83.2) | 60617684 (82.2) |  |
| CVD (%) | No | 55494033 (75.2) | 1233879 (83.5) | 0.002 |
|  | Yes | 18272222 (24.8) | 243732 (16.5) |  |
| CKD (%) | No | 1983 (0.1) | 23954696 (32.5) | 0.104 |
|  | Yes | 6252 (0.4) | 12797560 (17.3) |  |

Data are presented as frequencies (percentages) or mean (SD)

Abbreviations:

BMI, the body-mass index is determined as follows: the weight in kilograms (Kgs) / (height in square meters (m^2^)

CVD, cardiovascular disease

CKD, chronic kidney disease

## Table S2. Weighted baseline characteristics of participants in the different sex (weighted N=73772340).

|  | Level | Female | Male | p |
| --- | --- | --- | --- | --- |
| N |  | 41019469 | 32752871 |  |
| Age (years) |  | 71.0 (7.5) | 70.0 (7.2) | <0.001 |
| Race/ethnicity (%) | Non-Hispanic Black | 3792372 (9.2) | 2548834 (7.8) | <0.001 |
|  | Non-Hispanic White | 32415166 (79.0) | 26563675 (81.1) |  |
|  | Mexican American | 1432031 (3.5) | 1213486 (3.7) |  |
|  | Other Race | 3379901 (8.2) | 2426876 (7.4) |  |
| Education (%) | College or above | 17782181 (43.4) | 16676352 (50.9) | <0.001 |
|  | High school or equivalent | 11973018 (29.2) | 7621972 (23.3) |  |
|  | Less than high school | 11173343 (27.2) | 8409560 (25.7) |  |
| Marital status (%) | Never married | 1376515 (3.4) | 1162576 (3.5) | <0.001 |
|  | Living with partner | 493880 (1.2) | 660804 (2.0) |  |
|  | Married | 19983311 (48.7) | 24889289 (76.0) |  |
|  | Separated | 543697 (1.3) | 334527 (1.0) |  |
|  | Divorced | 4833751 (11.8) | 2619146 (8.0) |  |
|  | Widowed | 13788316 (33.6) | 3086530 (9.4) |  |
| Family income-poverty ratio |  | 2.66 (1.53) | 3.09 (1.54) | <0.001 |
| BMI (kg/m2) |  | 28.65 (6.37) | 28.48 (5.23) | 0.159 |
| Smoking status (%) | Never | 24372661 (59.4) | 10970775 (33.5) | <0.001 |
|  | Former | 12479206 (30.4) | 17382479 (53.1) |  |
|  | Now | 4146776 (10.1) | 4382876 (13.4) |  |
| Alcohol drinks (%) | Mild | 193833 (0.5) | 1356586 (4.1) | <0.001 |
|  | Moderate | 4437347 (10.8) | 2585936(7.9) |  |
|  | Heavy | 1222561 (3.0) | 2156038 (6.6) |  |
| Diabetes (%) | No | 31623339 (77.1) | 24087387 (73.5) | 0.002 |
|  | Yes | 9396129 (22.9) | 8665484 (26.5) |  |
| Hypertension (%) | No | 16239404 (39.6) | 14835027 (45.3) | <0.001 |
|  | Yes | 24780065 (60.4) | 17917844 (54.7) |  |
| Hyperlipidemia (%) | No | 6414037 (15.6) | 6738830 (20.6) | <0.001 |
|  | Yes | 34603643 (84.4) | 26014041 (79.4) |  |
| CVD (%) | No | 32339884 (78.8) | 23154149 (70.7) | <0.001 |
|  | Yes | 8676504 (21.2) | 9595719 (29.3) |  |
| CKD (%) | No | 12782811 (31.2) | 11171885 (34.1) | 0.003 |
|  | Yes | 7316357 (17.8) | 5481203 (16.7) |  |

Data are presented as frequencies (percentages) or mean (SD)

Abbreviations:

BMI, the body-mass index is determined as follows: the weight in kilograms (Kgs) / (height in square meters (m^2^)

CVD, cardiovascular disease

CKD, chronic kidney disease

Table S3. Weighted univariate cox regression model (weighted N=73772340)

|  | Level | HR | CI | P |
| --- | --- | --- | --- | --- |
| Age |  | 1.11 | 1.10-1.12 | 0.000 |
| Sex | Female | 1 |  |  |
|  | Male | 1.26 | 1.15-1.37 | 0.000 |
| Race/ethnicity | Mexican American | 1 |  |  |
|  | Non-Hispanic Black | 1.44 | 1.23-1.69 | 0.000 |
|  | Non-Hispanic White | 1.34 | 1.16-1.56 | 0.000 |
|  | Other Race | 1.11 | 0.89-1.39 | 0.360 |
| Education | College or above | 1 |  |  |
|  | High school or equivalent | 1.26 | 1.14-1.39 | 0.000 |
|  | Less than high school | 1.74 | 1.55-1.94 | 0.000 |
| Marital status | Never married | 1 |  |  |
|  | Living with partner | 0.78 | 0.51-1.20 | 0.258 |
|  | Married | 0.65 | 0.51-0.83 | 0.001 |
|  | Separated | 0.58 | 0.38-0.88 | 0.010 |
|  | Divorced | 0.82 | 0.64-1.05 | 0.109 |
|  | Widowed | 1.32 | 1.05-1.66 | 0.017 |
| Family income-poverty ratio |  | 0.80 | 0.77-0.82 | 0.000 |
| BMI (kg/m^2^) |  | 0.97 | 0.96-0.98 | 0.000 |
| Smoking status | Never | 1 |  |  |
|  | Former | 1.23 | 1.13-1.34 | 0.000 |
|  | Now | 1.58 | 1.41-1.78 | 0.000 |
| Alcohol drinks | Mild | 1 |  |  |
|  | Moderate | 1.11 | 0.74-1.65 | 0.618 |
|  | Heavy | 1.44 | 0.99-2.09 | 0.056 |
| Diabetes |  | 1.47 | 1.34-1.6 | 0.000 |
| Hypertension |  | 1.23 | 1.15-1.31 | 0.000 |
| Hyperlipidemia |  | 0.72 | 0.64-0.81 | 0.000 |
| CVD |  | 2.31 | 2.11-2.53 | 0.000 |
| CKD |  | 3.30 | 2.92-3.73 | 0.000 |

Abbreviations:

BMI, the body-mass index is determined as follows: the weight in kilograms (Kgs) / (height in square meters (m^2^)

CVD, cardiovascular disease

CKD, chronic kidney disease

Table S4 Weighted association between marital status and all-cause mortality for male in the multivariate and crude analyses (weighted N=32752871).

|  | Never married | Living with partner | Married | Separated | Divorced | Widowed |
| --- | --- | --- | --- | --- | --- | --- |
| Model1 | 1 | 0.73(0.45-1.17) | 0.56(0.40-0.79) | 0.81(0.44-1.50) | 0.86(0.60-1.25) | 1.45(1.03-2.05) |
| Model2 | 1 | 0.76(0.48-1.19) | 0.60(0.45-0.83) | 0.81(0.44-1.47) | 0.85(0.60-1.20) | 1.42(1.04-1.94) |
| Model3 | 1 | 0.95(0.60-1.52) | 0.79(0.56-1.10) | 0.88(0.48-1.62) | 0.96(0.68-1.35) | 1.54(1.09-2.17) |
| Model4 | 1 | 0.98(0.62-1.56) | 0.77(0.55-1.56) | 0.83(0.47-1.46) | 0.89(0.65-1.25) | 1.35(0.97-1.90) |

Data are hazard ratio (95% CI)

Model 1 unadjusted

Model 2 adjusted for age, ethnicity and education level

Model 3 adjusted for age, ethnicity, education level, family income-poverty ratio and BMI level

Model 4 adjusted for model 3 covariates as well as smoking status, alcohol drinks, diabetes, hypertension, hyperlipidemia, cardiovascular disease and chronic kidney disease

Abbreviations:

CI stands for confidence interval

BMI, the body-mass index is determined as follows: the weight in kilograms (Kgs) / (height in square meters (m^2^)

Table S5 Weighted association between marital status and all-cause mortality for female in the multivariate and crude analyses (weighted N=41019469).

|  | Never married | Living with partner | Married | Separated | Divorced | Widowed |
| --- | --- | --- | --- | --- | --- | --- |
| Model1 | 1 | 0.76(0.34-1.66) | 0.66(0.48-0.90) | 0.42(0.25-0.69) | 0.85(0.63-1.14) | 1.58(1.19-2.10) |
| Model2 | 1 | 0.74(0.33-1.65) | 0.66(0.48-0.92) | 0.42(0.25-0.70) | 0.86(0.63-1.18) | 1.51(1.11-2.05) |
| Model3 | 1 | 0.72(0.35-1.51) | 0.72(0.52-0.99) | 0.39(0.22-0.67) | 0.84(0.62-1.13) | 1.37(1.01-1.85) |
| Model4 | 1 | 0.55(0.24-1.25) | 0.68(0.50-0.94) | 0.35(0.20-0.59) | 0.79(0.60-1.04) | 1.17(0.87-1.58) |

Data are hazard ratio (95% CI)

Model 1 unadjusted

Model 2 adjusted for age, ethnicity and education level

Model 3 adjusted for age, ethnicity, education level, family income-poverty ratio and BMI level

Model 4 adjusted for model 3 covariates as well as smoking status, alcohol drinks, diabetes, hypertension, hyperlipidemia, cardiovascular disease and chronic kidney disease

Abbreviations:

CI stands for confidence interval

BMI, the body-mass index is determined as follows: the weight in kilograms (Kgs) / (height in square meters (m^2^)

Table S6 Weighted association between marital status and all-cause mortality for the three different age categories (weighted N=73772340).

| Age | Never married | Living with partner | Married | Separated | Divorced | Widowed |
| --- | --- | --- | --- | --- | --- | --- |
| 60-69 | 1 | 1.02(0.53-1.94) | 0.54(0.36-0.80) | 0.48(0.27-0.85) | 0.99(0.66-1.49) | 0.67(0.44-1.02) |
| 70-79 | 1 | 0.93(0.52-1.65) | 0.59(0.41-0.83) | 0.64(0.32-1.31) | 0.73(0.49-1.09) | 0.69(0.51-0.94) |
| ≥80 | 1 | 0.78(0.31-1.97) | 0.91(0.63-1.31) | 0.82(0.36-0.87) | 0.92(0.60-1.41) | 1.02(0.71-1.46) |

Data are hazard ratio (95% CI)

Figure S1. Kaplan–Meier curves of marital status for female and male


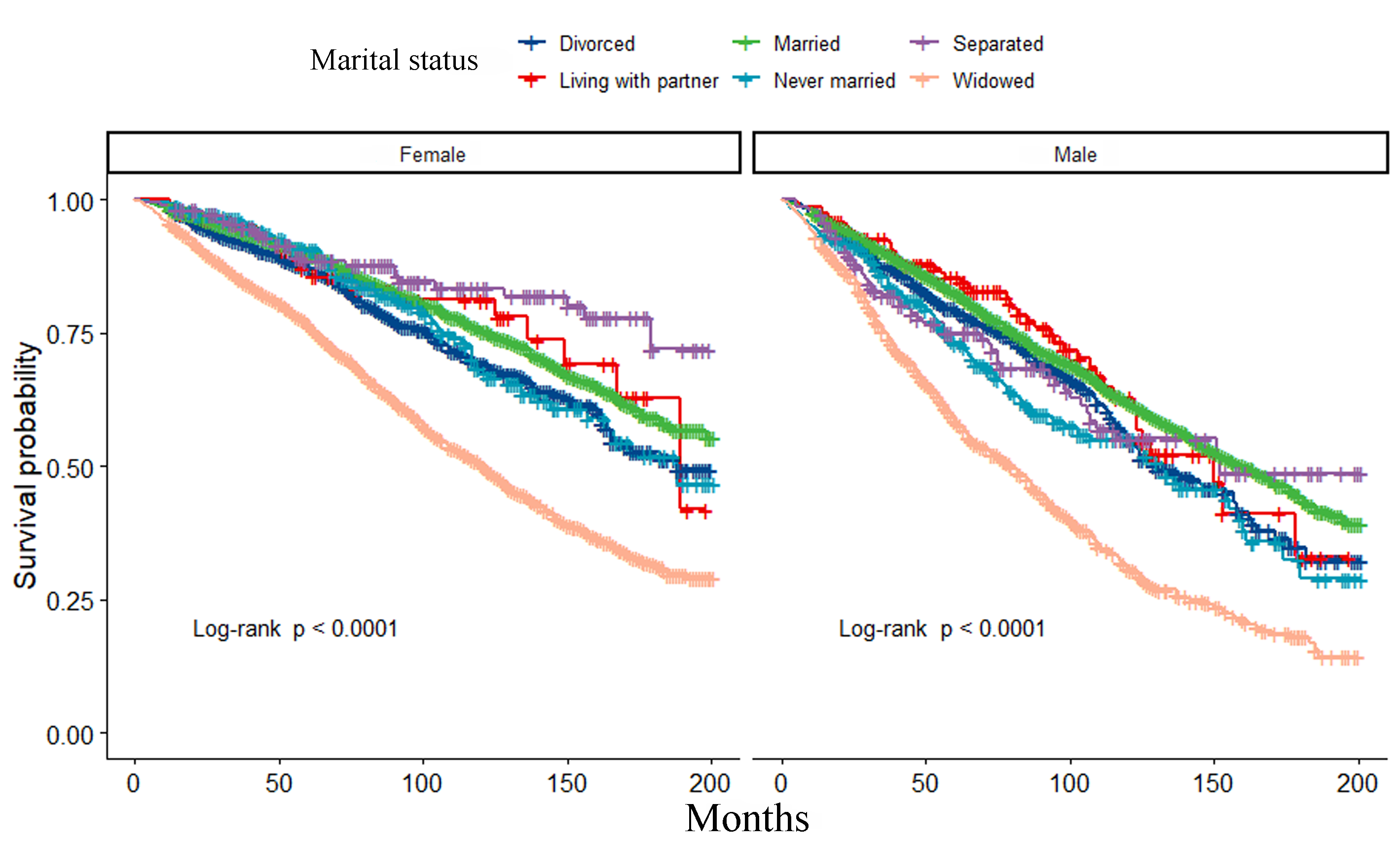

Supplement: Supplementary file 1 — Table S1. Weighted baseline characteristics of participants: marital status and marital status missing (weighted N = 75249951). Table S2. Weighted baseline characteristics of participants in the different sex (weighted N = 73772340). Table S3. Weighted univariate cox regression model (weighted N = 73772340). Table S4 Weighted association between marital status and all-cause mortality for male in the multivariate and crude analyses (weighted N = 32752871). Table S5 Weighted association between marital status and all-cause mortality for female in the multivariate and crude analyses (weighted N = 41019469). Table S6 Weighted association between marital status and all-cause mortality for the three different age categories (weighted N = 73772340). Figure S1. Kaplan?Meier curves of marital status for female and male. [file 12877_2023_3880_MOESM1_ESM.doc]
